# Ozone and pulsed electro-magnetic field therapies improve endometrial lining thickness in frozen embryo transfer cycles: Three case reports

**DOI:** 10.1097/MD.0000000000016865

**Published:** 2019-08-23

**Authors:** Zaher Merhi, Rajean Moseley-LaRue, Amber Ray Moseley, André Hugo Smith, John Zhang

**Affiliations:** a Department of Biochemistry, Albert Einstein College of Medicine Bronx; b New Hope Fertility Center, New York, NY; c HOCATT USA LLC, Weatherford, TX.

**Keywords:** frozen embryo transfer, infertility, IVF, ozone, PEMF, thin endometrial lining

## Abstract

**Rationale:**

In assisted reproductive technology, a persistently thin endometrial lining represents a huge challenge during frozen embryo transfer (FET) cycles.

**Patient concerns:**

Three patients who had a persistently thin endometrial lining despite the use of several medical agents known to improve endometrial lining thickness.

**Diagnoses:**

Infertility undergoing FET cycles.

**Interventions:**

A combination of transdermal and intravaginal ozone therapy along with Pulsed Electro-Magnetic Field (PEMF) therapy.

**Outcomes:**

Ozone with PEMF, both of which are known to have vasodilatatory, anti-inflammatory, and anti-oxidant actions, were successful in improving the thickness of the endometrial lining in all 3 patients. Two out of 3 patients became pregnant following single embryo transfer.

**Lessons:**

Ozone with PEMF constitute a novel experimental approach for women with persistently thin endometrial lining undergoing FET. This novel approach needs validation by large well-designed studies.

## 1 Introduction

In assisted reproductive technology (ART), a receptive endometrium with an appropriate threshold thickness is essential for successful embryo implantation and ongoing pregnancy.^[1,2]^ For instance, several studies have shown that an endometrial lining thickness (EMT) <7 mm was found to have a significantly lower implantation rate compared to an EMT of ≥7 mm.^[1,2]^ In frozen embryo transfer (FET) cycles, the EMT is traditionally measured by a transvaginal ultrasound in the follicular phase of a natural FET cycle or after preparation with estradiol treatment in medicated FET cycles.

Despite all efforts, a small percentage of patients undergoing FET do not develop thick enough EMT to proceed with the embryo transfer procedure. Several treatment protocols for inadequately thin endometrium include hormonal manipulation by estradiol (oral, vaginal, transdermal, or intramuscular),^[3]^ vasoactive agents such as low-dose aspirin,^[4]^ tocopherol (vitamin E),^[5]^ pentoxifylline,^[5]^ vaginal sildenafil,^[6]^ and others such as tamoxifen,^[7]^ granulocyte-colony-stimulating factor (G-CSF),^[8]^ and stem cell therapy.^[9]^ Despite these measures, many patients do not respond to any (or a combination) of these treatments. Here we report a case series of 3 patients undergoing FET with refractory thin EMT and who developed a satisfactory EMT following ozone sauna therapy (OST) using the Hyperthermic Ozone and Carbonic Acid Transdermal Technology (HOCATT).

## 2 Methods

### 2.1 Case report (s)

All patients have signed a written informed consent for publication of this case series. Ethical board approval by New England IRB was obtained (IRB# 120180241).

### 2.2 Patient #1

A 36-year-old patient with iatrogenic premature ovarian insufficiency presented with her partner to our clinic with a desire to conceive using donor oocyte. She had a history of well-controlled type-1 diabetes for which she was on insulin pump and metformin pills. Her last menstrual cycle was 3 years prior to her presentation to our clinic, and she had a history of dilatation and curettage for termination of pregnancy when she was 29 years old. She was a previous cigarette smoker and drank alcohol only socially. The male partner had a semen analysis that showed normal parameters except for a low concentration (4.5 Million/ml). A sonographic evaluation showed small ovaries and a normal uterus with EMT of 3 mm. Hysteroscopic evaluation of the uterus was performed and the cavity was found to be normal.

During a mock cycle evaluating the response of her lining to exogenous hormones, the patient received oral estradiol (E2) 4 mg daily that was increased to 8 mg, then changed to estradiol valerate intramuscularly twice weekly for a total of 4 weeks with poor response of the endometrium (maximal EMT reached 5 mm) despite a high serum E2 level of 692 pg/ml. Then oral tamoxifen 20 mg for a total of 5 days, daily baby aspirin (81 mg), vitamin E (1000 IU), and pentoxifylline (800 mg) were started but the lining dropped to 3.4 mm. Vaginal sildenafil 100 mg daily for a total of 2 weeks was added with minimal improvement of the EMT reaching only 5 mm. Thus the mock cycle was cancelled and the couple was extensively counseled about the experimental OST. Following a signed consent, the patient underwent 2 sessions of OST per week per week for a total of 3 weeks during which she was receiving E2 of 8 mg daily. Her lining finally improved to 7 mm after which vaginal progesterone 800 mg (prometrium) daily was started. On the 6th day of progesterone, FET of a blastocyst embryo (donor oocyte with her husband sperm) confirmed to be euploid by preimplantation genetic screening (PGS) was performed and it resulted in a single intrauterine pregnancy. The patient continued oral E2, which was stopped at 9 weeks of gestation and vaginal progesterone hormonal support was stopped at 12 weeks of gestation. An ultrasound at 10 weeks showed a normal intrauterine pregnancy with normal fetal heart rate. She was discharged from our clinic and she followed-up with her obstetrician. Her pregnancy is progressing normally at the time of writing this manuscript.

### 2.3 Patient #2

A 38-year-old healthy patient with severely diminished ovarian reserve was undergoing FET using donor oocyte. She did not have any significant medical, surgical, or social history. The male partner was healthy with normal semen analysis parameters. A sonographic evaluation showed small ovaries and a normal uterus with EMT of 3.5 to 4 mm. Hysteroscopic evaluation of the uterus was performed and the cavity was found to be normal. During a mock cycle evaluating the response of her lining to exogenous hormones, the patient received oral estradiol (E2) 4 to 8 mg, oral tamoxifen 20 mg for a total of 7 days, daily baby aspiring (81 mg), vitamin E (1000 IU), vaginal sildenafil 100 mg daily for 10 days, and pentoxifylline (800 mg) but the lining reached 4.5 mm in maximal thickness. Following a signed consent, the patient underwent 2 sessions of OST for a total of 3 weeks during which she was receiving E2 of 4 mg daily. Her lining reached 6.7 mm after which vaginal progesterone 800 mg daily was started. On the 6th day of progesterone, FET of a non-PGS tested blastocyst embryo was performed and it resulted in a single intrauterine pregnancy. At 6 weeks gestation, an ultrasound showed a missed abortion for which she underwent medical termination with vaginal misoprostol.

### 2.4 Patient #3

A 37-year-old healthy patient was undergoing FET using her own euploid embryos. She did not have any significant medical or social history, and her male partner was healthy with normal semen analysis parameters. Prior to her presentation to us, she had undergone hysteroscopy with dilatation and curettage for menorrhagia at an outside medical center. A sonographic evaluation showed normal size ovaries and a normal uterus with endometrial lining of 2 to 3 mm with areas where EMT was close to 0 mm. Hysteroscopic evaluation of the uterus was performed and the cavity was found to be normal with no scarring or adhesions.

Similar to patient #1 and #2, during a mock cycle evaluating the response of her lining to exogenous hormones, the patient received oral estradiol (E2) 4 to 8 mg, oral tamoxifen 20 mg for a total of 10 days, daily baby aspirin (81 mg), vitamin E (1000 IU), vaginal sildenafil 100 mg daily for a total of 10 days, and pentoxifylline (800 mg) but the lining reached 4 mm in maximal thickness. Following a signed consent, the patient underwent 2 sessions of OST per week for a total of 3 weeks during which she was receiving E2 of 4 mg daily. Her lining finally made it to 6.7 mm after which vaginal progesterone 800 mg daily was started. On the 6th day of progesterone, a frozen thaw transfer of a euploid blastocyst embryo was performed but she did not get pregnant.

### 2.5 The procedure: Ozone Sauna Therapy (OST) protocol using the Hyperthermic Ozone and Carbonic Acid Transdermal Technology (HOCATT)

The HOCATT is a machine that delivers ozone transdermally (ozone 1 setting at 500 ml/PM in our patients) and vaginally (ozone 2 setting at 200 ml/PM in our patients) via a silicone tube. It includes a chamber, an ozone generator for cabin ozonization, an ozone generator for simultaneous auxiliary ozone applications, a CO_2_ administration device and electric CO_2_ regulator for a CO_2_ cylinder. For vaginal ozone application, the patient herself introduces vaginally (around 1–2 inches internally) a very thin disposable catheter that infuses ozone while sitting inside the machine. The HOCATT infuses CO_2_ gas into the chamber at 5 L/PM, which will then mixes with the steam (H_2_O) in order to form Carbonic Acid (H_2_CO_3_). Carbon far infrared ray (FIR) pads (set at 100%), together with steam, are used to raise body temperature. It also contains a heart rate monitor, an oxygen humidifier container for pure oxygen breathing (at 2.5 L/PM) and a Pulsed Electro-Magnetic Field (PEMF) stand.

The participant gets completely undressed and sits in the sauna machine, with the head out in order to avoid breathing the ozone. Before each OST session, the blood pressure and heart rate are measured. The ozone concentration varies from 50% to 80% depending on the participant's tolerability. Each participant would ideally have 2 sessions per week for 3 weeks (total of 6 sessions). Each session is approximately 30 minutes. The first session starts with 50% ozone concentration which is then increased to 60% ozone concentration in the second session, 70% ozone concentration in the third session, and 80% ozone concentration in the fourth, fifth and sixth sessions.

## 3 Discussion

Ozone is a trivalent (O_3_) form of oxygen (O_2_). Ozone, a highly reactive molecule, is a potent oxidant and anti-inflammatory agent.^[10]^ It also has strong bactericidal, antiviral, anti-fungal, and anti-protozoal actions.^[10]^ Clinically, ozone therapy has been used to treat several pathologies such as cancers,^[11]^ chronic wounds,^[12]^ psoriasis,^[13]^ arthritis,^[14]^ eye disorders,^[15]^ osteomyelitis,^[16]^ musculoskeletal disorders,^[17]^ neurologic disorders,^[18]^ and many others. OST has historically been given transdermally, intraarterially, subcutaneously, intramuscularly, by autohemotransfusion, and even rectally.^[19]^ Transdermal ozone is a method by which ozone is introduced into the body via the skin while sitting in a hot steam cabinet while the head is out of the cabinet in order to prevent ozone inhalation. As the pores of the skin open as a result of being surrounded by the warm steam, ozone by-products partially enter the body transdermally. The ozone then penetrates the blood, lymph, and fat allowing ozone in and toxins out via the induced sweating process. The side effects of OST are rare but involve detox reaction, which includes flu-like symptoms, muscle aches, nausea, vomiting, headache, sneezing, and skin rash. OST is contraindicated in children, elderly, fever, any medical condition that interferes with the health of the female patient such as uncontrolled diabetes, uncontrolled hypertension, hypotension, cardiac disease, liver disease, renal disease, any malignancy, ozone allergy or intolerance, hematological disorders, stroke, myocardial infarction, thyrotoxicosis, glucose-6-phosphate dehydrogenase (G6PD) deficiency, acute alcohol intoxication, excessive caffeine intake, heat insensitivity, intake of medications that impair sweating, hypoglycemia, dehydration, organ transplant patients, cutaneous porphyria, vitiligo, seizures, electrical implants, uncontrolled dyslipidemia, and COPD.^[20]^

Some of the mechanisms for its therapeutic potentials include the generation of peroxides by ozonolysis with unsaturated fatty acids in cell membranes, activation or generation of reactive oxygen species (ROS) which function as physiological enhancers of various biological processes (including increased production of ATP), and increased expression of intracellular enzymes with antioxidant activity.^[20]^ Additionally, ozone improves blood flow and the release of O_2_ from hemoglobin into the tissues^[21]^; for example, in patients with occlusive peripheral arterial disease, the reinfusion of autologous blood after ozonization in a glass box can correct the altered hemostatic-hemorheological parameters such as platelet aggregation, whole blood viscosity, and erythrocyte filterability.^[21,22]^ Another study in sheep showed that ozone induces a dose-dependent increase in bronchial artery flow, which is the result of a vasodilatation of the bronchial vasculature.^[23]^

The HOCATT machine delivers ozone both transdermally and vaginally using the manufacturer's protocol. In addition to ozone, the HOCATT machine delivers PEMF, which is a biophysical therapeutic modality based on the delivery of pulsed electromagnetic fields. PEMF can have a vasodilatatory (microcirculatory)^[24,25]^ and anti-inflammatory effects.^[26]^ The vasodilator effect of PEMF could be mediated by increasing nitric oxide (NO), a well-known vasodilator.^[27]^ This effect has been shown to enhance microvascular perfusion and tissue oxygenation in rat brain by demonstrating that 30 minutes of PEMF treatment induced cerebral arteriolar dilation leading to an increase in microvascular blood flow and tissue oxygenation that persisted for at least 3 hours.^[24]^ In clinical studies, PEMF therapy has been associated with improvement in postoperative pain,^[28,29]^ most likely via an anti-inflammatory action.^[30]^ Using human cells (human dermal fibroblasts, human epidermal keratinocytes, and human mononuclear cells) in culture,^[26]^ PEMF treatment showed significant changes in the relative amount of mRNA encoding enzymes involved in heme catabolism and removal of ROS, including an increase in heme oxygenase 1 and superoxide dismutase 3, and a relative increase in mRNA encoding enzymes involved in lipid mediator biosynthesis, including an increase in arachidonate 12- and 15-lipoxygenase mRNA.^[26]^ Additionally, PEMF treatment showed a decrease in the mRNA levels of several cytokines such as interleukin 1β.^[26]^ These results suggest that PEMF therapy may promote the resolution of chronic inflammation by mediating cellular gene expression changes.

In this case series, 1 patient had a successful pregnancy, the second patient had a miscarriage (albeit unknown whether aneuploidy was responsible for this because the embryo was not tested by PGS), and the third patient did not get pregnant despite achieving a relatively thick EMT. The EMT is usually correlated with clinical pregnancy rate, which is a pregnancy that is confirmed by both elevated levels of hCG and ultrasound confirmation of a pregnancy. A clinical pregnancy includes a missed abortion or a stillbirth. Given this definition, 2 out of 3 patients presented in this manuscript had a clinical pregnancy. In patient #3, even though she had a euploid embryo transferred and a relatively thick EMT following OST, she did not get pregnant. However, it is well known that a euploid embryo has approximately a 65% chance of producing a clinical pregnancy despite thick EMT. The 35% probability of not achieving a clinical pregnancy with a euploid embryo is still not understood. Finally, the treatment of persistently thin EMT remains a major clinical challenge among fertility patients and is very frustrating for both the patient and the physician. Despite all the experimental treatment protocols, a large percentage of those women continue to have a thin EMT, which frequently leads to using a gestational carrier as a last resort. The limitations of this study include the small sample size of participants and the lack of a control group; thus large studies pertaining to OST and thin EMT are clearly needed in order to support our preliminary findings. Future studies should include randomized trials with a control group that has not been exposed to OST. However, this case series lays the ground for OST as a novel experimental approach that could be offered for women who have persistently thin EMT due to previous injury to the endometrium by surgeries such as dilatation and curettage or cesarean section, women with chronic endometritis, or women who has low uterine blood flow.

## Author contributions

**Conceptualization:** Zaher Merhi, John Zhang.

**Investigation:** Zaher Merhi, André Hugo Smith, John Zhang.

**Methodology:** Zaher Merhi, Rajean Moseley-LaRue, Amber Ray Moseley, André Hugo Smith.

**Resources:** Amber Ray Moseley, John Zhang.

**Supervision:** Zaher Merhi.

**Writing – original draft:** Zaher Merhi.

**Writing – review & editing:** Zaher Merhi, Rajean Moseley-LaRue, Amber Ray Moseley, André Hugo Smith, John Zhang.
